# Efficacy of non-invasive neuromodulation for chronic tinnitus: a systematic review and meta-analysis of randomized controlled trials

**DOI:** 10.1007/s00405-026-10541-0

**Published:** 2026-08-20

**Authors:** Mohamed Mohamed Elmoursy, Waleed Mohammed Nemr Mohamed

**Affiliations:** Audiovestibular Medicine unit, ENT Department, Al-Azhar University, Assuit, Egypt

**Keywords:** Tinnitus, Neuromodulation, Transcranial magnetic stimulation, Transcranial direct current stimulation, Tinnitus Handicap Inventory (THI)

## Abstract

**Background:**

Chronic tinnitus affects approximately 14% of adults worldwide, with nearly 2% experiencing severe, persistent symptoms that impair daily functioning. Non-invasive neuromodulation techniques, such as repetitive transcranial magnetic stimulation (rTMS), transcranial direct current stimulation (tDCS), and acoustic or nerve stimulation, have emerged as potential therapies, yet their clinical effectiveness remains uncertain. This systematic review and meta-analysis synthesized evidence from randomized controlled trials (RCTs) evaluating the efficacy of neuromodulation for chronic tinnitus.

**Methods:**

PubMed, Scopus, and Web of Science were searched through September, 2025. Eligible studies were RCTs assessing non-invasive neuromodulation versus sham in adults with chronic tinnitus. The primary outcome was change in Tinnitus Handicap Inventory (THI) scores. Secondary outcomes included the Tinnitus Functional Index (TFI), Tinnitus Questionnaire (TQ), loudness measures, and anxiety and depression scales.

**Results:**

A total of 32 trials comprising 1,561 participants were included. Neuromodulation significantly improved tinnitus severity at short-term, with a pooled THI reduction of − 6.96 points (95% CI − 10.67 to − 3.26). rTMS showed a significant benefit (MD − 4.35, 95% confidence interval [CI] − 7.36 to − 1.33), as did acoustic/nerve stimulation (MD − 8.70, 95% CI − 15.81 to − 1.60), whereas tDCS demonstrated a nonsignificant effect (MD − 9.14, *p* = 0.10). Benefits were most pronounced immediately post-treatment and at 1–3 months, with effects diminishing by 6 months (MD − 3.93, *p* = 0.06). Secondary outcomes showed no significant improvements in TFI, TQ, objective loudness, or mood scales. Subjective loudness on visual analogue scale (VAS) showed a small borderline improvement (standardized mean difference [SMD] − 0.38, 95% CI − 0.75 to 0.00).

**Conclusion:**

Non-invasive neuromodulation provides modest, clinically meaningful short-term reduction in tinnitus severity, particularly with rTMS at dorsolateral prefrontal cortex area and acoustic/nerve stimulation. However, effects are inconsistent across modalities and decrease over time. Future studies should prioritize standardized protocols, optimized stimulation parameters, and personalized targeting to enhance therapeutic efficacy.

**Supplementary information:**

The online version contains supplementary material available at https://doi.org/10.1007/s00405-026-10541-0.

## Introduction

Chronic tinnitus is typically defined as the perception of ringing or buzzing sounds in the ears in the absence of an external source that persists for longer than six months [1, 2]. It is a common, debilitating condition, affecting approximately 14% of the adult population, corresponding to an estimated 740 million individuals with a higher incidence in older age. About 2% of people experience severe, persistent tinnitus that markedly diminishes quality of life [3, 4]. Tinnitus patients usually experience significant distress, insomnia, concentration difficulties, and psychiatric comorbidities such as anxiety and depression, contributing to marked economic burdens [5]. Despite this impact, there is currently no definitive cure or reliably effective medical treatment for chronic tinnitus.

In most cases, tinnitus is thought to arise from maladaptive neuroplastic changes in the central auditory system, often triggered by cochlear damage or hearing loss [2]. Reduced auditory input may lead to a compensatory increase in central neuronal gain and hyperactivity in auditory pathways, manifesting as a “phantom” sound perception. Neuroimaging and neurophysiological studies have shown abnormal spontaneous activity and synchronized firing in the auditory cortex and associated neural structures in tinnitus patients [2, 6]. Moreover, non-auditory brain regions including limbic, frontal, and salience networks, appear to be recruited in tinnitus pathology, contributing to the persistent awareness of the sound and the related emotional distress [7]. Accordingly, interventions that directly modulate cortical excitability or disrupt pathological neural synchrony might help attenuate the tinnitus percept. Neuromodulation therapies such as, transcranial magnetic stimulation (TMS) and transcranial direct current stimulation (tDCS) are designed to externally modulate brain activity [8].

Repetitive transcranial magnetic stimulation (rTMS) is a non-invasive technique that uses pulsating magnetic fields to induce electrical currents in specific brain regions, thereby modulating neural activity [9]. In tinnitus treatment, rTMS is typically applied at the auditory cortex or sometimes the prefrontal cortex, with the goal of recalibrating the hyperactive neural circuits underlying the phantom sound [10]. The tDCS approach, in turn, delivers a weak direct electrical current between scalp electrodes to subtly alter cortical excitability with modulation of the tinnitus-generating network. Hence, both rTMS and tDCS are attractive from a safety perspective and they directly address the brain activity alterations believed to sustain tinnitus, providing a strong theoretical rationale for their use in this context [11, 12].

Over the past two decades, these neuromodulation methods have been increasingly tested in adults with chronic tinnitus and several recent studies and reviews have evaluated the clinical efficacy of rTMS and tDCS for chronic tinnitus, although findings remain mixed and the evidence for tDCS efficacy in tinnitus is inconclusive some patients appear to experience relief under certain stimulation conditions, but results across studies have varied widely with limited insights about long-term efficacy of neuromodulation [12–15]. Therefore, this systematic review and meta-analysis investigated the efficacy of different approaches of neuromodulation in adult patients with chronic tinnitus.

## Methods

### Protocol and registration

Our systematic review and meta-analysis were conducted in compliance with PRISMA statement guidelines [16] and the instructions outlined in the Cochrane Handbook of Systematic Reviews and Meta-Analysis of interventions (version 5.1.0) [17]. We registered our protocol on Prospero with the following registration number: CRD420251230250.

### Eligibility criteria

We included randomized controlled trials (RCTs) published in English that met the following predefined PICO eligibility criteria:

- Population: Adult patients aged 18 years or older diagnosed with unilateral or bilateral chronic tinnitus.
- Intervention: Different approaches of non-invasive neuromodulation including rTMS, tDCS and other nerve stimulation
- Comparator: Sham neuromodulation
- Outcomes: Our primary outcome was assessment of tinnitus severity using Tinnitus Handicap Inventory (THI) at different time points. Secondary outcomes focused on Tinnitus Functional Inventory (TFI), and Tinnitus Questionnaire (TQ). In addition, we assessed perceived loudness using Visual Analog Scale (VAS) and objective loudness. Moreover, depression and anxiety related to tinnitus were assessed using Beck Depression Inventory (BDI) and Beck Anxiety Inventory (BAI).

#### Exclusion criteria

Studies were excluded if they were reviews, editorials, commentaries, conference abstracts, animal studies, lacked efficacy data, or published in languages other than English.

### Data sources and search strategy

A detailed literature search was conducted for studies published up to September, 2025 in different electronic databases including PubMed, Scopus, and Web of Science using the following search strategy (“tinnitus” OR “subjective tinnitus” OR “chronic tinnitus”) AND (“neuromodulation” OR “transcranial magnetic stimulation” OR rTMS OR “transcranial direct current stimulation” OR tDCS OR “acoustic neuromodulation” OR “deep brain stimulation”). Detailed search strategy for each database is shown in Table S1 in supplementary file.

### Study selection and data extraction

#### Screening and selection process

We blindly screened all titles and abstracts for potentially relevant articles using Rayyan website [18]. Then, full-text assessment of potentially eligible articles was performed according to the eligibility criteria.

#### Data extraction

For each included study, we manually extracted detailed data using a structured form. We extracted a summary of each study, which included the following items: Study ID, study design, location, sample size, population criteria and follow-up duration. Demographic data and characteristics of such as age, sex, laterality of tinnitus and its duration were also collected. Moreover, the details of neuromodulation approach involving type of neuromodulation, duration of intervention, frequency and number of sessions were extracted in separate sheet.

### Assessment of risk of bias

We independently evaluated the risk of bias assessment of the included studies. The quality assessment of RCTs was assessed using the revised Cochrane risk-of-bias tool (RoB 2) [19]. Each of the following domains randomization process, deviations from intended interventions, missing outcome data, outcome measurement, and selection of reported outcomes was separately rated as "low risk," "high risk," or "some concerns".

### Statistical analysis

The meta-analysis was performed using Comprehensive Meta-Analysis software (CMA v.3) [20] and Review Manager (RevMan) software [21] on the extracted outcome data that present in at least 3 of the included studies. Regarding continuous outcomes, data was pooled as mean difference (MD) or standardized mean difference (SMD) with 95% confidence interval (CI) due to different outcome reporting scales. The level of statistical significance was set to be *p* < 0.05. A random effect model (Inverse variance) was adopted rather than a fixed effect model yielding a more conservative estimate of the pooled effect and generalizable results. Heterogeneity was evaluated using the chi-square test with a significance level of P ≤ 0.1 and an I^2^ value greater than 40%. We place greater emphasis on the I^2^ value, as it performs better than the chi-square test when the number of included studies is small. Subgroup analysis was conducted where applicable. Publication bias was assessed visually and by statistical tests. Finally, we conducted meta-regression for the primary outcome on duration of illness.

## Results

### Search results

**Fig. 1:**
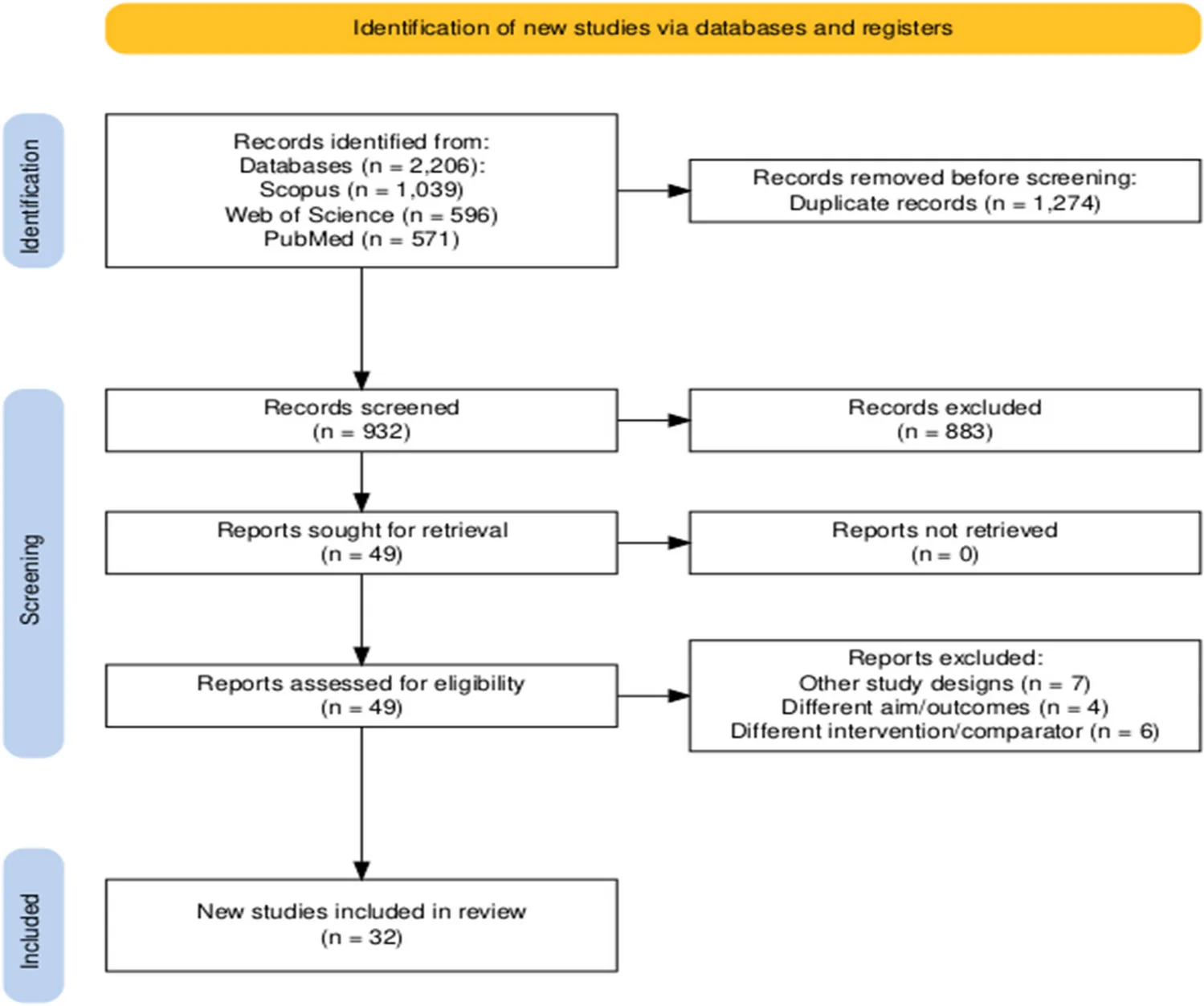
PRISMA flow diagram of included studies

The literature search across Scopus, PubMed, and Web of Science identified 2,206 records. After removing 1,274 duplicates, the remaining 932 records underwent title and abstract screening which resulted in exclusion of 883 ineligible records. Forty-nine full texts were assessed according eligibility criteria and seventeen studies were excluded with reasons. Thirty-two studies [22–52] met inclusion criteria and entered the qualitative synthesis and meta-analysis (Fig. 1).

### Characteristics of studies

Across 32 clinical trials published between 2010 and 2024, conducted across Europe, Asia, and North America, 14 studies used rTMS, 13 studies adopted tDCS, and the remaining 5 trials applied acoustic or nerve stimulation in adults with chronic subjective tinnitus of different duration compared to sham intervention. Outcome assessment focused on change in THI scores. Follow-up periods and assessment endpoints ranged between immediately post-intervention, at 1 month, 3 months, and after 6 months or longer. The eligible studies involved 914 participants received active neuromodulation approaches and 647 received sham neuromodulation. Summary of studies and characteristics of participants are shown in Table 1. Detailed protocols of neuromodulation including type of intervention and duration are shown in Table 2

**Table 1:** Summary of included studies and patient characteristics

Study ID	Location	Population	Sample Size		Duration of Tinnitus, months, mean (SD)		Age, mean (SD)		Male, n (%)		Bilateral Tinnitus, n (%)		Follow-up dura tion
			Intervention	Control	Intervention	Control	Intervention	Control	Intervention	Control	Intervention	Control	
Anders 2010	Czech Republic	Adults aged 18 to 70 years naïve with regards to rTMS, with unilateral or bilateral tinnitus according to ICD-10 of at least 6 months duration	22	20	106.8 (81.6)	88.4 (67.5)	48.09 (14.86)	50.05 (13.97)	12 (54.54)	17 (85)	13 (59.1)	14 (70)	26 weeks
Aydin 2020	Turkey	Patients with subjective tinnitus for at least six months	40	22	24.67 (20)	29 (28.53)	49.47 (14.16)	49.31 (12.72)	24 (60)	10 (45.5)	11 (27.5)	8 (36.3)	1 month
Bilici 2015	Turkey	Adult patients with unilateral or bilateral moderate-to-severe tinnitus for at least 1 year	75		> 1 year		40 (13.2)		33 (44)		36 (48)		6 months
Bolandi 2024	Iran	Adult patients with unilateral or bilateral subjective tinnitus with no history of Meniere’s disease	17	17	22.88 (14.55)	21.12 (13.43)	37.26 (7.1)		NR	NR	12 (70.6)	13 (76.47)	1 month
Cardon 2022	Belgium	Adult patients with tinnitus of 6 months to 50 years	38	39	124.32	105.48	53.47 (14.39)	51.95 (13.87)	20 (52.6)	23 (58.9)	17 (44.7)	11 (28.21)	8 (2 weeks)
Chung 2012	Taiwan	Adult patients with chronic tinnitus for at least 6 months	12	10	87	69	53.83 (14.8)	51.9 (15.5)	11 (91.7)	9 (90)	3 (25)	2 (20)	1 month
Emadi 2023	Iran	Adult patients with chronic tinnitus for at least 6 months	17	17	34.53 (30.06)	28.53 (22.23)	44.65 (9.62)	47.82 (10.81)	22 (64.7)		NR		1 month
Formanek 2018	Czech Republic	Adult patients suffering from unilateral or bilateral chronic subjective non-pulsatile primary tinnitus for at least 6 months	20	12	53.4 (61.89)	76.8 ± 76.85	47.9 (14.31)	51.8 (10.34)	13 (65)	10 (83.33)	27 (84.4)		6 months
Garin 2011	Belgium	Adult patients aged 18–80 years, with tinnitus of at least 2 months with stable hypoacousia	20		1 year to more than 10 years		50.9 ± 12.9		16 (80)		10 (50)		8 weeks
Hall 2022	United Kingdom	Adult patients of both sexes with chronic subjective tinnitus for more than 3 months	50	50	NR	NR	49.1(11.3)	51.8 (12.2)	33 (66)	37 (74)	NR	NR	36 weeks
Han 2024	Republic of Korea	Adult patients aged 18 years or older who had suffered from subjective tinnitus for over 3 months	1 session: 10, 2 sessions: 11	9	> 3 months		1 session: 46.62 (12.03), 2 sessions: 44.11 (9.31)	47.14 ± 9.14	9 (42.9)	4(44.44)	7(33.33)	3(33.33)	8 weeks
Hoekstra 2013	Netherlands	Patients with a non-fluctuating tinnitus of at least 2 months duration	26	24	57.9 (58)	127 (102)	50 (12)	55 (12)	26(100)	15(63)	20 (77)	10(42)	6 months
Jones 2023	United States (USA)	Adult participants (aged 18 years) with bothersome somatic tinnitus	49	50	more than 6 months		47 (12.7)		59 (60)				3 months
Landgrebe 2017	Germany	Adult patients of both sexes, aged 18–70 years with chronic tinnitus defined as a duration of at least 6 months	71	75	74.2	97.2	48.1 (12.5)	49.9 (13.2)	54(76.1)	51 (68)	113 (77.4)		26 weeks
Lee 2014	South Korea	Patients diagnosed with subjective, unilateral tinnitus and followed up for longer than 6 months	45	20	24.44 (19.79)		46.6 (13.9)	45.6 (11.5)	26 (57.78)	13 (65)	0	0	NR
Li 2022	China	Adult patients (18 < 70 years old), with subjective tinnitus as the first clinical complaint	30	22	19.2	21.6	36.4 ± 11.7	39.9 (10.2)	14 (46.7)	14 (63.6)	NR	NR	2 weeks
Marcondes 2010	Brazil	Patients with uni- or bilateral tinnitus of at least 3 months duration, age over 18 years and normal pure tone audiometry (thresholds less than 25 dB)	10	9	> 3 months		NR	NR	NR	NR	NR	NR	6 months
Mares 2022	Czech Republic	Patients aged at least 18 years of age with a history of tinnitus lasting at least 6 months	19	20	48.47 (57.6)	33.2 (33.42)	49 (16.73)	46.15 (18.5)	11 (57.89)	12 (60)	NR	NR	6 months
Martins 2024	Brazil	Patients older than 18 years old; with chronic tinnitus symptom with a duration of at least three months, occurring at least 5 min a day on most days	24	18	> 3 months		44.64 (15.13)	52 (14.65)	10 (41.7)	8 (44.44)	NR	NR	1 month
Nikakhlagh 2023	Iran	Adult patients with reported a chronic tinnitus lasting for at least 12 months	21	21	97.7	79.44	46.38 (7.16)	46.19 (9.23)	13 (61.9)	9 (42.86)	13 (30.95)		2 weeks
Noh 2020	South Korea	Adult patients with chronic essential tinnitus for more than 6 months	16	16	76.1 (129.3)	85.1 (102.4)	54.1 (9.4)	55.9 (8.8)	13 (50)	11 (68.75)	5 (31.3)	5 (31.3)	12 weeks
Pal 2015	Switzerland	Adults with chronic (1 year) non-pulsatile subjective tinnitus and age-adjusted normal hearing according to the Presbycusis Scale	21	21	63.1 (64.9)	71 (102.3)	51.6 (12.2)	48 (9.9)	12 (57.1)	12 (57.1)	21 (100)	21 (100)	3 months
Yilmaz 2014	Turkey	Patients aged between 15 and 70 years old with tinnitus for at least 6 months	30	30	> 6 months		49.8 (8.03)	NR	27 (45)	NR	24 (40)	NR	1 month
Souza 2020	Brazil	Adults with unilateral or bilateral chronic tinnitus	12	12	23 (17.61)	54.25 (100)	44.58 (16.20)	50.50 (9.72)	2 (16.67)	6 (50)	2 (16.67)	5 (41.67)	5 days
Tutar 2020	Turkey	Adult patients with non-pulsatile, subjective tinnitus complaints for > 3 months	40	20	31 (49)	NR	41.17 (10.75)	NR	22 (36.7)	NR	20 (50)	NR	1 month
Yadollahpour 2024	Iran	Adult patients aged 18–65 years with chronic and medications resistant tinnitus (THI ≥ 38) with disease duration of more than 2 years	25	15	89.76	91.2	47.52 (7.51)	47.67 (7.96)	22 (45.8)	NR	3 (7.5)	NR	1 month
Shekhawat 2014	New Zealand	Participants with chronic tinnitus (more than 2 years), aidable hearing loss with no previous experience of hearing aid use	20	20	19.78	16.55	59.85 (9.6)	58.5 (6.4)	18 (90)	18 (90)	NR	NR	6 months
Sahlsten 2017	Finland	Patients with chronic (6 months–10 years), uni- or bilateral tinnitus in the age group of 18–65 years	19	20	64.8	58.8	48.9 (13.1)	51.5 (10.7	13 (68.4)	14 (70)	11 (57.9)	13 (65)	6 months
Plewnia 2012	Germany	Adult outpatients with a less than 5 years history of chronic subjective tinnitus	16	16	28 (13)	22 (14)	55.8 (9.7)	45.6 (10.3)	7 (43.75)	8(50)	6 (37.5)	9 (56.25)	12 weeks
Leaver 2024 (2)	United States (USA)	Participants were between 18 and 75 years old, and had chronic tinnitus for at least one year	10	10	30.9 (22.19)	45.1 (15.15)	43.53 (13.17)	39.50 (13.97)	4 (40)	4 (40)	9 (90)	8 (80)	1 month
Abtahi 2018	Iran	Patients suffering from tinnitus for at least 1 year, aged between 18 and 80 years	Anodal group: 23, cathodal = 23	23	NR	NR	A: 47.4 (15.8), C: 46.9 (14.6)	45.4 (13.2)	A: 16 (69.6), C: 15(65.2)	15 (65.2)	NR	NR	NR
Folmer 2015	United States (USA)	Adult patients with chronic tinnitus	32	32	NR	NR	58.3(9.5)	62.8 (8.3)	25 (78.13)	26 (81.25)	NR	NR	26 weeks

*SD* standard deviation, *rTMS* repetitive transcranial magnetic stimulation, *NR* not reported

**Table 2:** Types of Neuromodulation modalities and treatment protocols

Study ID	Type of neuromodulation	Frequency (Hz)	Treatment sessions	Treatment duration per session	Stimulatory area
Anders 2010	rTMS	1 HZ	1500 pulses per session for 5 sessions over 5 days		Left auditory cortex
Aydin 2020	rTMS	1 HZ	1500 pulses per session for 5 sessions over 5 days	30 min	Left temporoparietal lobe
Bilici 2015	rTMS	1–10 HZ	600–900 pulses per session for 10 sessions over 14 days	15 min	Left temporoparietal lobe
Bolandi 2024	tAVNS	0.1 to 5 mA	daily session for 6 sessions	20 min	the left ear
Cardon 2022	tDCS	2 mA	6 sessions over 3 weeks	15 min for each area (total: 30 min)	left temporal area and right dorsolateral prefrontal cortex
Chung 2012	rTMS	50 Hz	900 pulses daily session over 10 consecutive business days		Auditory cortex
Emadi 2023	tDCS	2 mA	6 sessions of tDCS every other day	20 min	Right dorsolateral prefrontal cortex
Formanek 2018	rTMS	1–25 Hz	2300 pulses per session for 5 sessions over 5 days		Auditory and dorsolateral prefrontal cortex
Garin 2011	tDCS	1 mA	3 sessions with 2 weeks interval	20 min	Left temporoparietal area
Hall 2022	T30 Neurostimulator (acustic stimulation)	1.5 Hz	T30 neurostimulator device and headphones at visits		
Han 2024	tDCS	2 mA	one session or two session per day twixe a week for 1 months	20 min	Bifrontal cortex
Hoekstra 2013	rTMS	1 HZ	2000 pulses persession 5 sessions over 5 days		Auditory cortex
Jones 2023	Somatosensory and Auditory stimulation		Daily session over 5 weeks	30 min	Trigeminal ganglion or the C1 and C2 cervical spinal cord
Landgrebe 2017	rTMS	1 HZ	2000 pulses per session 10 sessions over 2 weeks		Left auditory cortex
Lee 2014	TENS	15 mA	Eight therapy sessions over 4 weeks	6.5 min	External ear pinna
Li 2022	rTMS	1 HZ	1000–2000 Pulses per session 10 sessions over 2 weeks		Temporoparietal lobe
Marcondes 2010	rTMS	1 HZ	Five sessions of rTMS were performed on five consecutive business days	17 min	Left temporoparietal cortex
Mares 2022	tDCS	1.5 mA	6 sessions over 2 weeks	20 min	Dorsolateral prefrontal cortex
Martins 2024	tDCS	2 mA	5 sessions for five consecutive day sessions	20 min	Left temporoparietal area
Nikakhlagh 2023	tDCS	2 mA	24 sessions over 1 month	20 min	Auditory cortex and the dorsolateralprefrontal cortex
Noh 2020	rTMS	1 HZ	2000–3000 pulses per session for 4 sessions over 4 days		Left auditory and prefrontal cortex or left auditory cortex
Pal 2015	tDCS	1 mA	5 sessions over 5 days	20 min	Auditory and prefrontal cortex
Yilmaz 2014	rTMS	1 HZ	10 sessions over 10 days	30 min	
Souza 2020	tDCS	2 mA	Five tDCS sessions during a period of 5 consecutive days	20 min	Dorsolateral prefrontal cortex
Tutar 2020	TENS	1–200 Hz	10 sessions over 1 month	30 min	Cymba conchae (auricular branch of the vagus nerve)
Yadollahpour 2024	tDCS	2 mA	5 sessions over 5 days	20 min	Dorsolateralprefrontal cortex
Shekhawat 2014	tDCS with hearing aid	2 mA	5 consecutive sessions with 24-h gap	20 min	left temporoparietal area
Sahlsten 2017	rTMS	1 HZ	10 treatment sessions over 2 weeks		Left auditory cortex
Plewnia 2012	rTMS	50 HZ	2400 pulses per session 20 sessions over 1 month		Temporoparietal cortex
Leaver 2024	tDCS	2 mA	Sessions five consecutive days	20 min	Left auditory cortex
Abtahi 2018	tDCS	2 mA	Session of scalp	20 min	T3 and T4 on scalp according International 10–20 system
Folmer 2015	rTMS	1 HZ	2000 pulses per session for 10 sessions over 2 weeks		Auditory cortex

*rTMS* repetitive transcranial magnetic stimulation, *TENS* transcutaneous electrical nerve stimulation, *tAVNS* transcutaneous auricular vagus nerve stimulation, *tDCS* transcranial direct current stimulation

### Risk of bias and quality assessment

**Fig. 2:**
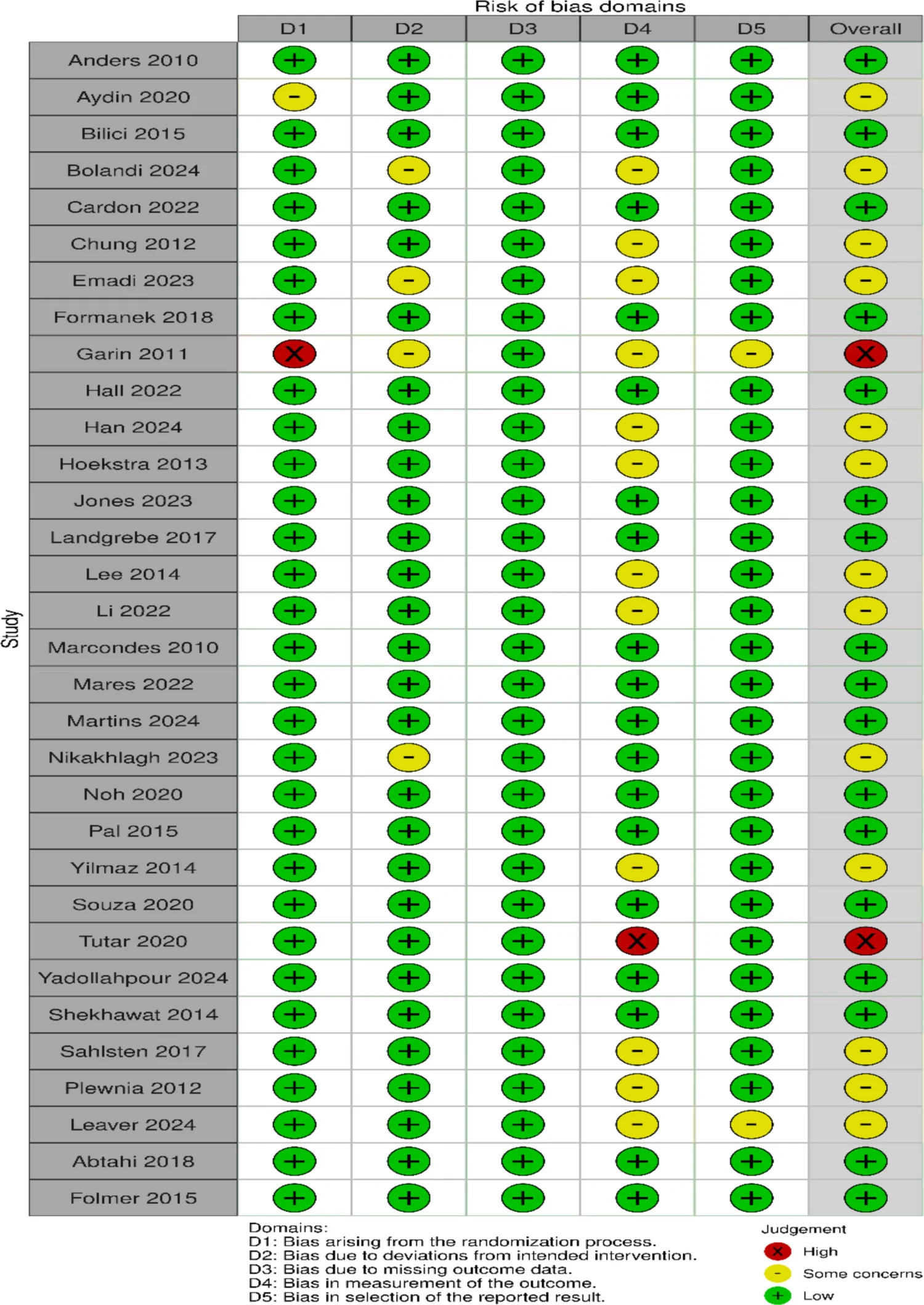
Risk of bias summary of the included RCTs

On evaluation the quality of included studies, the majority of included studies had good quality with low risk of bias. Thirteen studies showed overall some concerns due to unclear risk of bias across different domains especially deviation for intended protocol or outcome measurement. Two studies were rated as poor quality with high risk of bias due to randomization, blinding or outcome measurements. Figure 2 shows a summary of the risk-of-bias assessment.

### Primary outcomes

#### Tinnitus handicap inventory (THI)

**Fig. 3:**
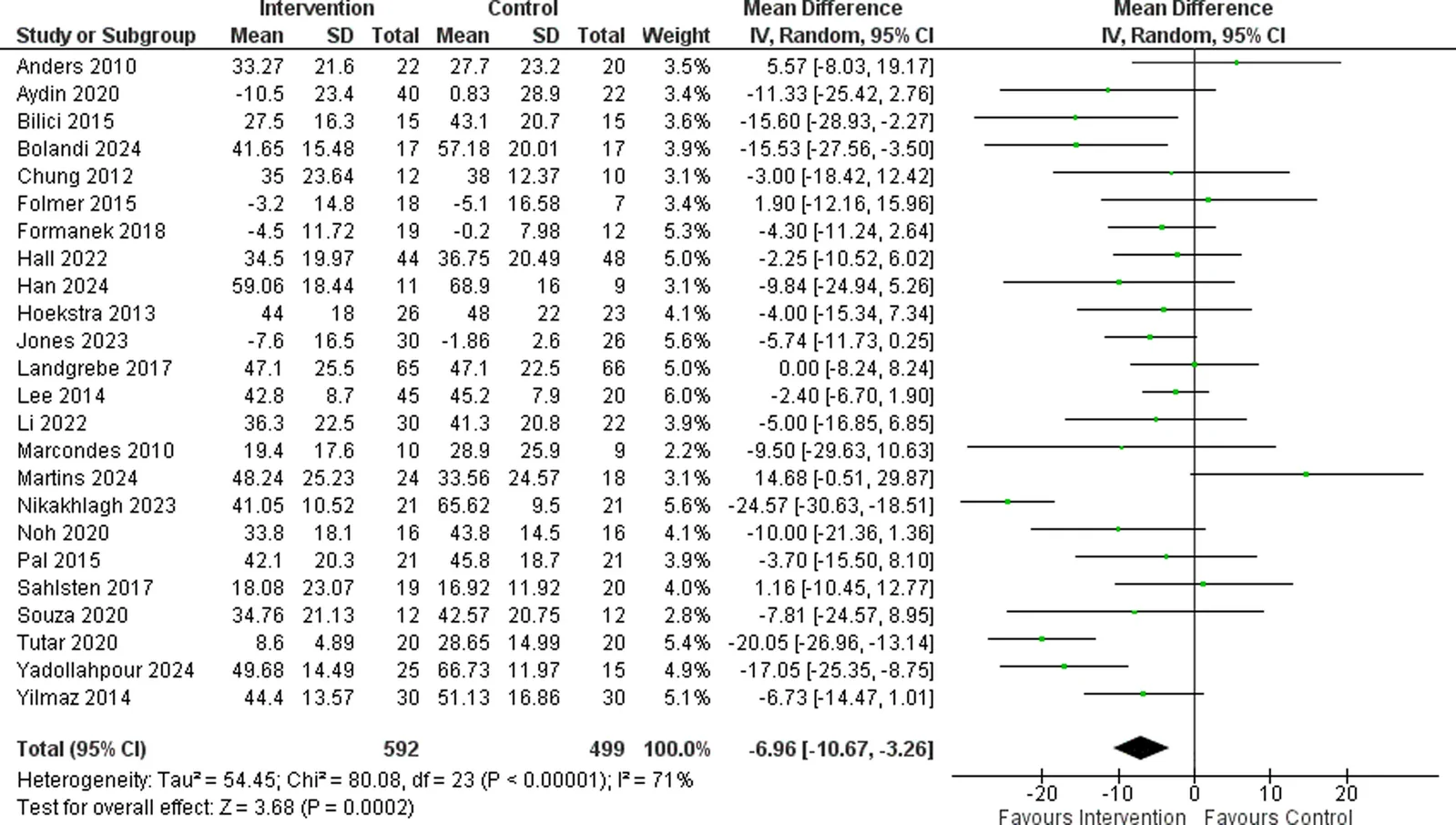
Tinnitus Handicap Inventory (THI) (Pooled analysis of all modalities)

**Fig. 4:**
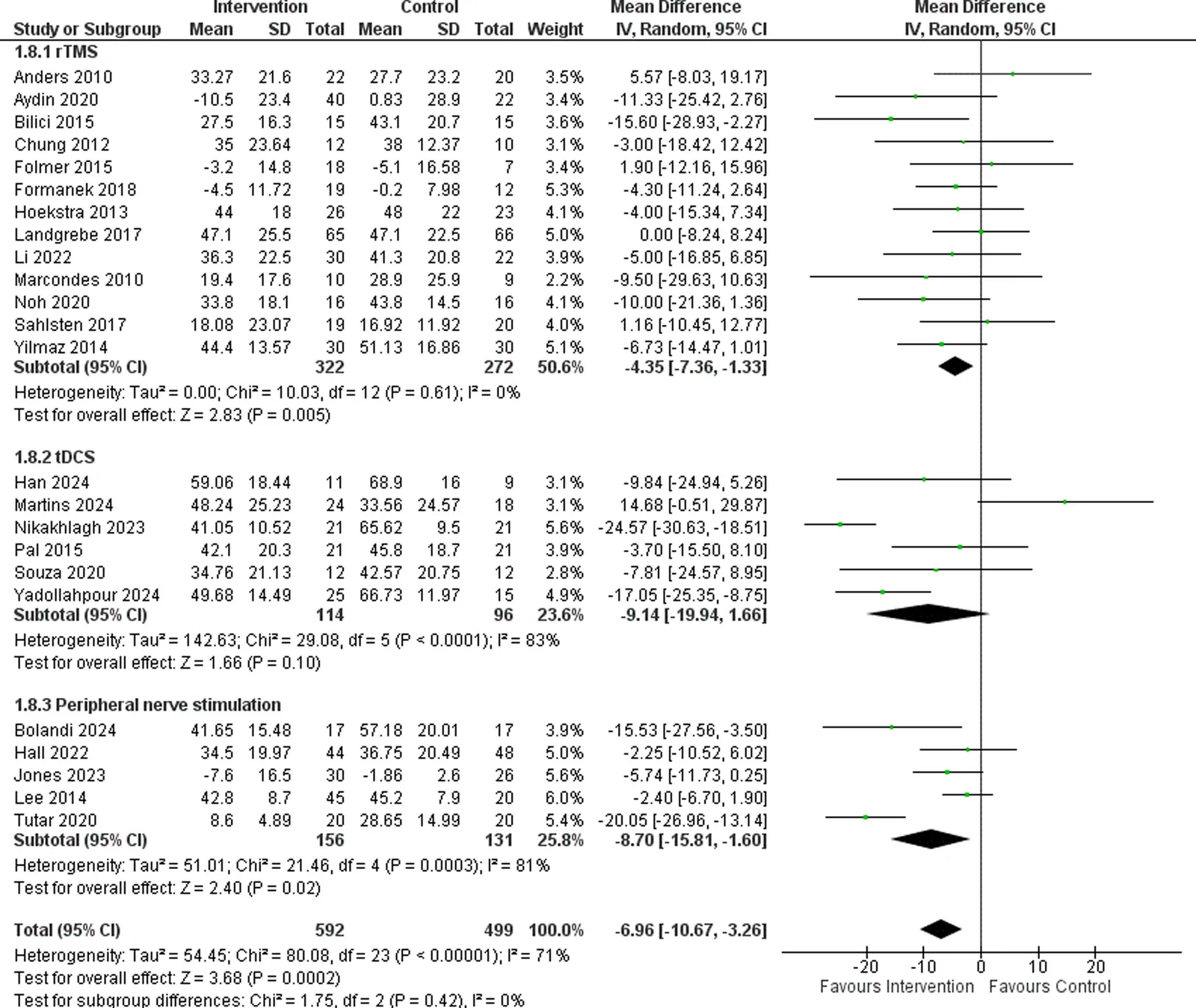
Tinnitus Handicap Inventory (THI) (Subgroup analysis according intervention)

**Fig. 5:**
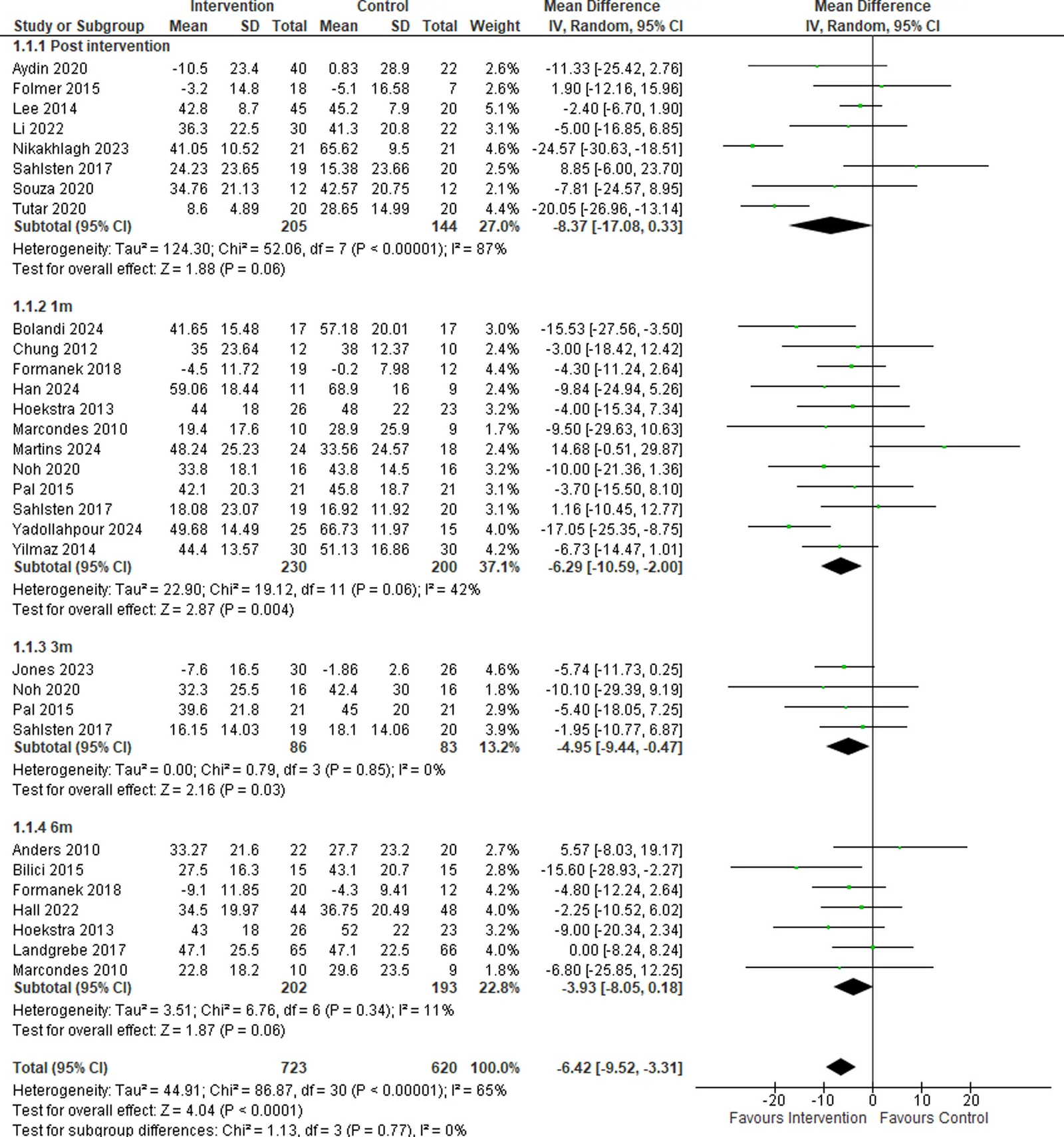
Tinnitus Handicap Inventory (THI) (Subgroup analysis according time of assessment)

Pooled random-effects analysis of total THI score favored neuromodulation over control (mean difference (MD: − 6.96, 95% CI: − 10.67 to − 3.26; *p* = 0.0002), with substantial between-study heterogeneity (*p* < 0.00001; I^2^ = 71%) (Fig. 3). By conducting subgroup analysis according to modality, rTMS showed a significant benefit (MD: − 4.35, 95% CI: − 7.36 to − 1.33; p = 0.005), acoustic stimulation also favored active treatment (MD: − 8.70, 95% CI: − 15.81 to − 1.60; *p* = 0.02), while tDCS demonstrated a nonsignificant difference (MD: − 9.14, 95% CI: − 19.94 to 1.66; *p* = 0.10) (Fig. 4). On stratifying studies according to time of assessment, effects were largest immediately post-intervention (MD: − 8.37, 95% CI: − 17.08 to 0.33; *p* = 0.06) and at 1 month (MD: − 6.29, 95% CI: − 10.59 to − 2.00; *p* = 0.004), remained significant at 3 months (MD: − 4.95, 95% CI: − 9.44 to − 0.47; *p* = 0.03), and attenuated by 6 months (MD: − 3.93, 95% CI: − 8.05 to 0.18; *p* = 0.06) (Fig. 5). Tests for subgroup differences across time points were not significant, and the across-time pooled estimate remained beneficial (MD: − 6.42, 95% CI: − 9.52 to − 3.31; *p* < 0.0001).

**Fig. 6:**
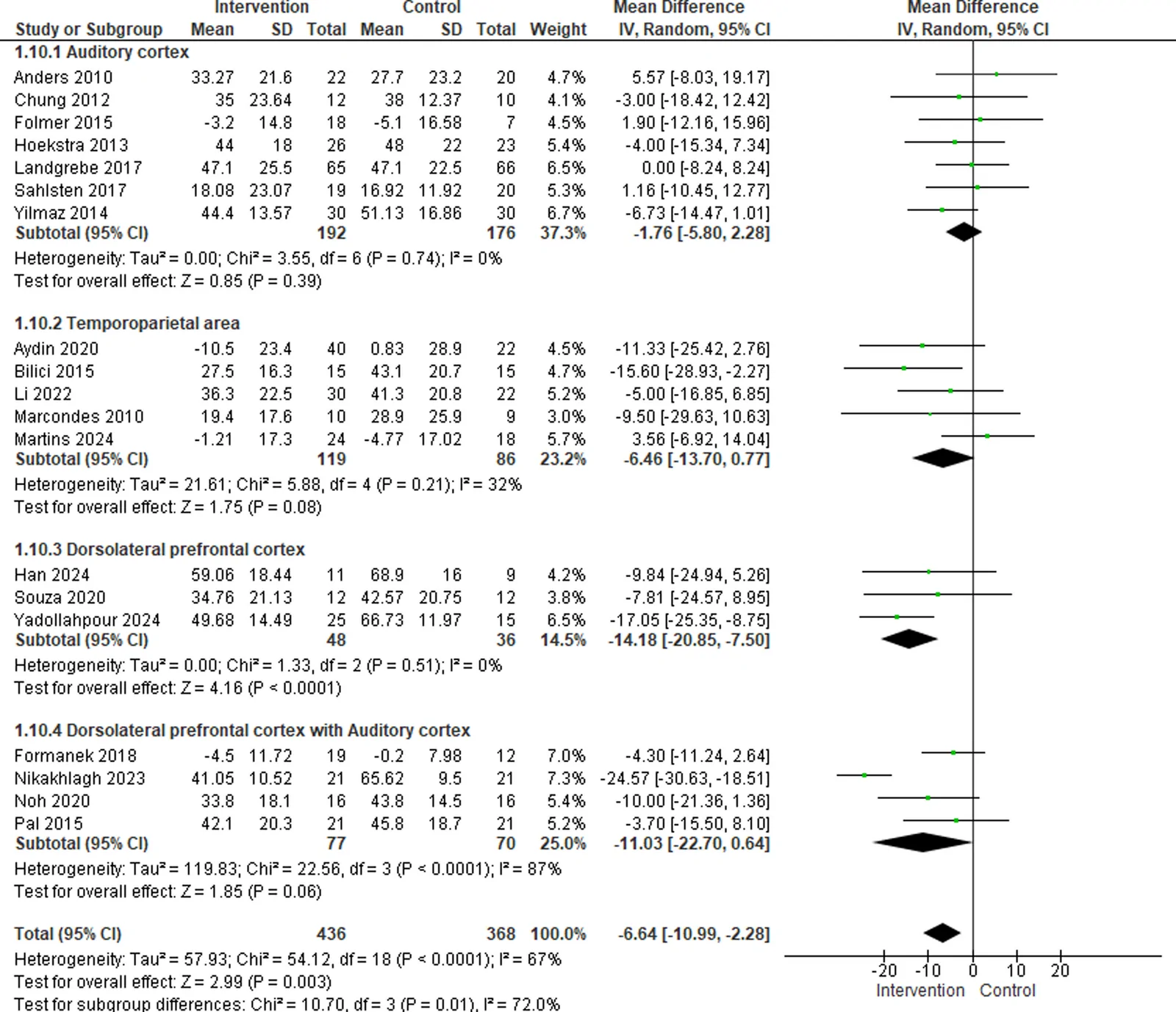
Tinnitus Handicap Inventory (THI) (Subgroup analysis according to stimulated cortical area)

In the subgroup analysis based on stimulated cortical area, neuromodulation targeting the auditory cortex showed no significant benefit compared with control (MD: − 1.76, 95% CI − 5.80 to 2.28; I^2^ = 0%). Stimulation of the temporoparietal area demonstrated a trend toward improvement, but this did not reach statistical significance (MD: − 4.66, 95% CI − 13.70 to 0.77; p = 0.08). In contrast, dorsolateral prefrontal cortex (DLPFC) stimulation was associated with a significant reduction in tinnitus severity (MD − 14.18, 95% CI − 20.85 to − 7.50; *p* < 0.0001; I^2^ = 0%). Combined stimulation of the DLPFC and auditory cortex showed a nonstatistically significance with substantial heterogeneity despite notable difference (MD − 11.03, 95% CI − 22.70 to 0.64; I^2^ = 87%). Overall, neuromodulation significantly improved tinnitus outcomes compared with control (MD − 6.64, 95% CI − 10.99 to − 2.28), with moderate heterogeneity (I^2^ = 67%), and a significant difference was observed between subgroups (*P* = 0.01), (Fig. 6).

**Fig. 7:**
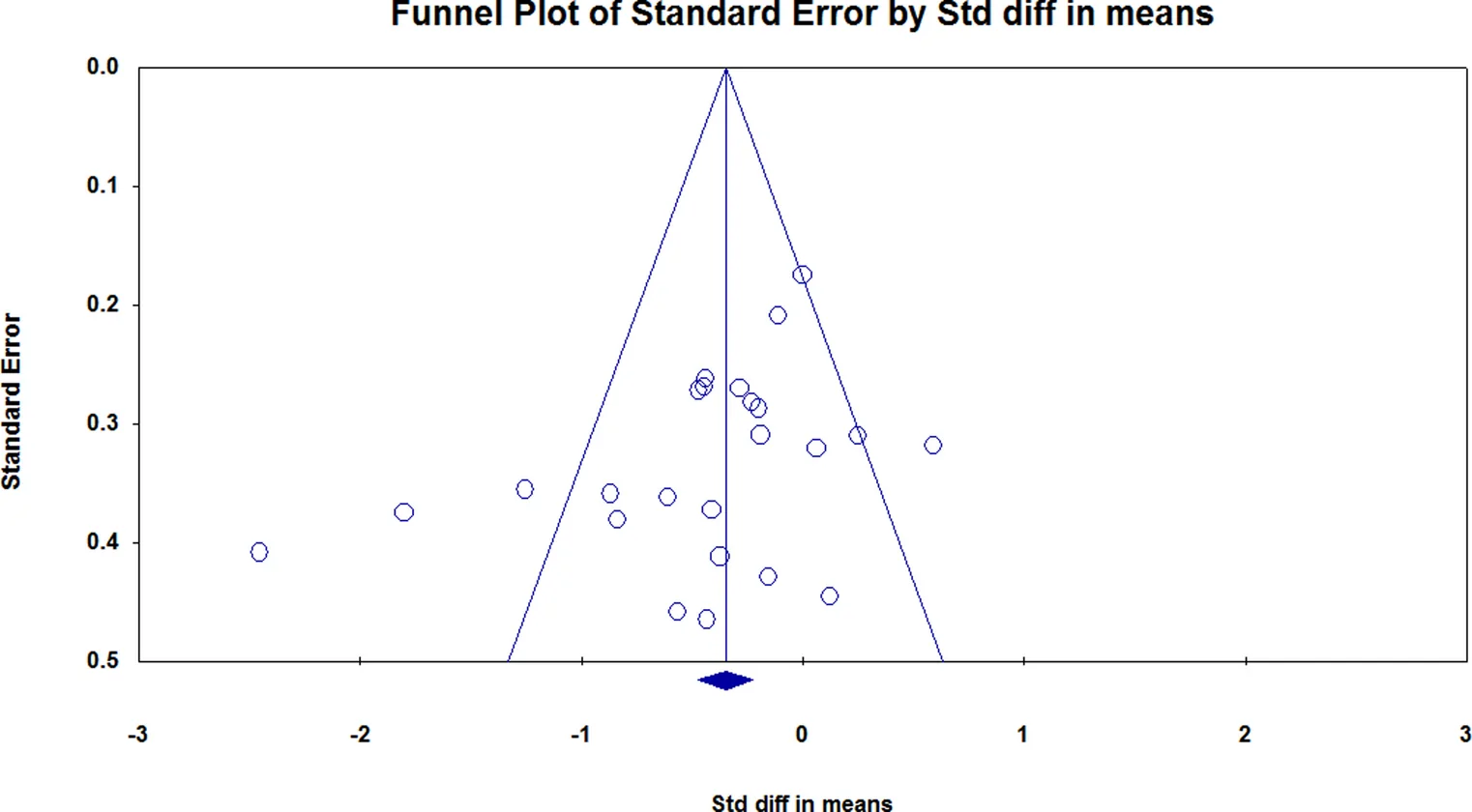
Funnel plot of publication bias of THI

Visual and quantitative tests provided mixed signals but indicated that any small-study effects have little impact on the pooled estimate (Fig. 7). Begg–Mazumdar rank correlation suggested no significant asymmetry (*p* = 0.15). In contrast, Egger’s regression intercept was positive and statistically significant (*p* = 0.043), consistent with some asymmetry. Duval and Tweedie’s trim-and-fill imputed six studies to the left of the mean; under the random-effects model this moved the standardized mean difference only trivially toward the null (from about − 0.66 to − 0.65), leaving the confidence interval and direction of effect essentially unchanged (Figure S1). Overall, while Egger’s test suggests the presence of small-study effects, the trim-and-fill and fail-safe analyses indicate that publication bias, if present, is unlikely to overturn the benefit observed for neuromodulation on THI.

In a restricted maximum likelihood exploratory meta-regression including 14 studies, tinnitus duration reported in months was not associated with treatment effect (Beta coefficient ≈ 0.0000, SE 0.0061; 95% CI: − 0.0120 to 0.0119; *p* = 0.9945) (Figure S2). The model explained essentially none of the between-study variance (R^2^ analog ≈ 0%), and substantial residual heterogeneity persisted (I^2^ = 73.3%; Q = 44.88, *p* < 0.001).

### Secondary outcomes

#### Tinnitus functional inventory (TFI) and tinnitus questionnaire

Tinnitus Functional Index evaluated across 7 studies showed no effect (MD = 0.17, 95% CI: − 2.40 to 2.73; I^2^ = 17%; *p* = 0.90) (Figure S3), and the pooled analysis of Tinnitus Questionnaire suggested a nonsignificant trend favoring neuromodulation (MD = − 3.61, 95% CI: − 7.98 to 0.77; I^2^ = 38%; *p* = 0.11) (Figure S4).

#### VAS and loudness

Regarding tinnitus loudness rated on a visual analogue scale reported in 14 studies, active neuromodulation yielded a small improvement that reached the threshold of statistical significance (SMD = − 0.38, 95% CI: − 0.75 to 0.00; *p* = 0.05), with considerable heterogeneity (I^2^ = 79%) (Figure S5). Additionally, objective loudness measured in dB in ten studies showed no difference versus control (MD = − 0.38, 95% CI: − 3.46 to 2.70; I^2^ = 74%; *p* = 0.81) (Figure S6).

#### Anxiety and depression scales

Pooled analysis of mood outcomes especially depression and anxiety were similarly non-significant. BDI analysis MD of − 2.33 compared to sham intervention with 95% CI: − 7.63 to 2.96; (*p* = 0.39) and BAI (MD = − 2.06, 95% CI: − 10.48 to 6.36; I^2^ = 89%; *p* = 0.63) (Figure S7 and S8 respectively).

## Discussion

Tinnitus is thought to arise from maladaptive neuroplastic changes and pathological synchronous neural activity in auditory pathways and connected brain regions. Chronic tinnitus is associated with hyperactivity and altered oscillatory rhythms in the auditory cortex, in addition to dysfunctional connectivity involving non-auditory areas such as dorsal lateral prefrontal cortex, or limbic system that modulate attention and emotional responses to sound [2, 53]. Neuromodulation therapies aim to reversibly modulate neural excitability and synaptic plasticity in these networks to disrupt the tinnitus network and reducing the salience or intensity of the phantom perception [54]. However, the evidence regarding clinical efficacy of neuromodulation is debatable. Therefore, our comprehensive systematic review and meta-analysis bring together current knowledge about different non-invasive approaches neuromodulation in alleviating symptoms of chronic tinnitus.

Based on our analysis, we observed that non-invasive neuromodulation therapies provide a modest but significant reduction in tinnitus severity as measured by the THI. Pooled analysis of all modalities revealed that neuromodulation was associated with an average THI improvement of approximately 7 points compared to sham (MD = –6.96). Notably, 6–7 points change could achieve minimal clinically important difference for the total THI score, indicating that the observed clinical and statistically significant benefit in the short-term [55, 56]. Although it showed modest improvement, this change may be perceptible for adult patients.

On conducting subgroup analysis for tinnitus severity using THI according to type of neuromodulation modalities, we found significant benefits for rTMS with approximately MD of –4.35 in addition to nerve and acoustic stimulation approaches (MD = –8.70) compared to sham-intervention. Regarding tDCS, it showed reduction in THI total score of about 9.14 points, however, it did not reach statistical significance compared to sham intervention. These findings are consistent with a systematic review and meta-analysis by Lefebvre-Demers et al., who noted that rTMS significantly reduced tinnitus symptoms compared to sham intervention, however, tDCS did not show significant difference [12].

Notably, the therapeutic benefits of different neuromodulation approaches were related to time of assessment after intervention since clinical gains were notable at the end of the treatment period and within the period from 1–3 months post-intervention. However, the pooled effect of neuromodulation approach at long-term after 6 months did not significantly differ from sham intervention (MD: − 3.93, *p* = 0.06). These insights are consistent with a meta-analysis by He et al., who reported non-significant benefits of rTMS after six months despite notable effects in tinnitus symptoms reduction after 1 month [13]. Notably, the pooled analysis according to stimulated cortical area showed that DLPFC had the significant reduction in THI scores with over 14 points reduction and consistent findings across studies. This finding suggests that incorporating prefrontal neuromodulation improves outcomes compared to auditory cortex targeting alone. Recent studies reported that left DLPFC produced significantly additional greater improvement in THI over follow-up period, whereas auditory cortex only stimulation showed no therapeutic effect [43, 44, 52]. Moreover, this insight aligns with the current concept supporting the role of DLPFC stimulation in tinnitus loudness reduction and providing an add-on benefit when combined with auditory cortex stimulation [57]. A recent network meta-analysis further supports the relative efficacy of interventions involving DLPFC compared with several auditory cortex approaches alone [58].

Regarding severity assessment of tinnitus symptoms, we observed no clinically meaningful improvements in broader functional assessments measured by TFI and Tinnitus Questionnaire scores compared to control groups. Additionally, we evaluated long-term effect of neuromodulation on mood and anxiety symptoms as tinnitus is chronic disease which negatively impacts quality of life and daily activities [59, 60]. However, our findings demonstrated non-significant difference between active and sham groups regarding anxiety and depression scores which aligns with current literature suggesting no significant effect of rTMS on tinnitus questionnaire or depressive symptoms on BDI [61]. On the other hand, neuromodulation approaches produced a small decrease in subjective pain related to tinnitus loudness measured using VAS (SMD = − 0.38, *p* = 0.05) with borderline statistical significance, but there were no significant changes in objective loudness measures such as audiometric loudness matching reported in dB. Hence, these findings suggest that neuromodulation can alleviate the perceived burden of tinnitus reflected in THI and self-reported loudness in the short-term, but its impact on objective tinnitus characteristics, general functional impairment, or comorbid psychological distress is limited with no statistical significance and related to objective assessment in many cases, indicating the need for further studies assessing neurophysiological aspects and quality of life in tinnitus patients.

### Current literature and limitations

Previous systematic reviews and meta-analysis studies were conducted to assess the clinical effects of neuromodulation modalities for tinnitus. Yin et al. [61], and He et al. [13], reported results which are comparable to our findings. However, these reviews were confined to only one modality (rTMS) with smaller number of included studies. Furthermore, Heiland et al. [62], conducted meta-analysis to evaluate effect of different neuromodulatory approaches in chronic subjective tinnitus, however, this analysis was restricted to of 19 RCTs. In this updated meta-analysis, we present 32 clinical trial investigating clinical impact of neuromodulation on tinnitus symptoms aiming to provide more conclusive evidence. Despite these insights, our study has some limitations. Heterogeneity was observed across outcomes which may be attributed to the variation in neuromodulation modalities, different, stimulating area, number of sessions, frequencies and intensity of electrical current in studies duration of tinnitus across studies. Additionally measuring tools used in pain assessment using VAS scares of ten points, while others used 100-point scale which may introduce inconsistency of results. To address the inconsistencies across studies, we conducted subgroup analysis according to stimulation approach (rTMS, tDCS and peripheral stimulation), stimulated cortical areas and different follow-up periods, aiming to provide comprehensive review about the role of different non-invasive neuromodulation approach in tinnitus and to guide the future research about the better modality, cortical region and endpoints in this population.

### Clinical implications, challenges and future directions

From a clinical overview, our findings confirm that neuromodulation therapies have a promising role in tinnitus management evidenced by the significant improvements in THI with reduction in the intrusiveness and functional impact of tinnitus severity following neuromodulatory treatment in the short-term. Moreover, using rTMS for tinnitus is still largely limited to research settings and require frequent visits for weeks, which may limit adherence and its application. Optimizing stimulating parameters form different neuromodulation modalities is still challenging since across included studies frequency such as, 1 and 10 Hz or higher frequencies in addition to time of each session and stimulated cortical areas, raising concerns about the need for standard protocol. Some recent studies have even tested personalized targeting using functional neuroimaging to guide coil positioning [63]. Additionally, combination approaches may yield synergistic benefits especially with preliminary evidence that sequentially combining tDCS and rTMS [64]. Similarly, combining neuromodulation alongside with traditional therapies for tinnitus may improve its symptoms.

## Conclusion

In conclusion, our findings highlight that non-invasive neuromodulation techniques hold promise as potential interventions for chronic tinnitus, particularly, on stimulating DLPFC in the short-term. While it demonstrated modest improvements in symptom reduction and quality of life, inconsistencies across trials suggest that standardized protocols. Future clinical trials with larger sample sizes are required to refine therapeutic targets and enhance reliability of findings.

## Supplementary information

Below is the link to the electronic supplementary material.

Supplementary file1 (DOCX 135 KB)

## Data Availability

The datasets used and/or analyzed during the current study are in the main manuscript.

## Abbreviations

BAI: Beck Anxiety Inventory
BDI: Beck Depression Inventory
CI: Confidence Interval
CMA: Comprehensive Meta-Analysis Software
MD: Mean Difference
PRISMA: Preferred Reporting Items for Systematic Reviews and Meta-Analyses
RCT: Randomized Controlled Trial
REML: Restricted Maximum Likelihood
RevMan: Review Manager Software
RoB 2: Revised Cochrane Risk of Bias Tool
rTMS: Repetitive Transcranial Magnetic Stimulation
SMD: Standardized Mean Difference
tDCS: Transcranial Direct Current Stimulation
DLPFC: Dorsolateral prefrontal cortex
TFI: Tinnitus Functional Index
THI: Tinnitus Handicap Inventory
TQ: Tinnitus Questionnaire
TENS: Transcutaneous Electrical Nerve Stimulation
VAS: Visual Analog Scale

## Declarations

### Clinical trial number

Not applicable because this was a study of publicly available, published data.
