# Supplementary material for: Efficacy of non-invasive neuromodulation for chronic tinnitus: a systematic review and meta-analysis of randomized controlled trials
Source: Eur Arch Otorhinolaryngol. 2026 Aug 20;283(11):7001–18. doi: 10.1007/s00405-026-10541-0 (PMC13748099; doi:10.1007/s00405-026-10541-0)
Supplement: Supplementary file 1 [file 405_2026_10541_MOESM1_ESM.docx]

**Supplementary materials**

Table S1. Search strategy for each database

| **Database** | **Search strategy** | **Number of records** |
| --- | --- | --- |
| **PubMed** | ("Tinnitus"[Mesh] OR tinnitus OR "subjective tinnitus" OR "chronic tinnitus")  AND  ("Neuromodulation" OR "Transcranial Magnetic Stimulation" OR "transcranial magnetic stimulation" OR rTMS OR "repetitive transcranial magnetic stimulation" OR "Transcranial Direct Current Stimulation" OR tDCS OR "acoustic neuromodulation" OR "Deep Brain Stimulation"[Mesh] OR "deep brain stimulation" OR DBS) | **571** |
| **Scopus** | TITLE-ABS-KEY (tinnitus OR "subjective tinnitus" OR "chronic tinnitus")  AND  TITLE-ABS-KEY (neuromodulat* OR "transcranial magnetic stimulation" OR rtms OR "repetitive transcranial magnetic stimulation" OR "transcranial direct current stimulation" OR tdcs  OR "acoustic neuromodulation" OR (acoustic W/3 neuromodulat*) OR "bimodal stimulation" OR (auditory W/3 stimulation) OR "deep brain stimulation" OR dbs) | **1039** |
| **Web of science** | TS= (tinnitus OR "subjective tinnitus" OR "chronic tinnitus")  AND  TS= (neuromodulation OR "transcranial magnetic stimulation" OR rTMS OR "repetitive transcranial magnetic stimulation" OR "transcranial direct current stimulation" OR tDCS OR "acoustic neuromodulation" OR "deep brain stimulation" OR DBS) | **596** |
| **Total** | | **2206** |

**Supplementary Figures**

**Supplementary Figure S1. Publication bias funnel plot (imputed)**

**Supplementary Figure S2. Meta regression analysis of association between THI and tinnitus duration**

**Supplementary Figure S3. Tinnitus Functional Inventory (TFI)**

**Supplementary Figure S4. Tinnitus Questionnaire**

**Supplementary Figure S5. VAS loudness**

**Supplementary Figure S6. Objective Loudness dB**

**Supplementary Figure S7. Beck Depression Inventory (BDI)**

**Supplementary Figure S8. Beck Anxiety Inventory (BAI).**
